# Racial Disparity in Healthcare Experience Among Women Seeking Fertility Care During the COVID-19 Pandemic

**DOI:** 10.1007/s40615-025-02372-2

**Published:** 2025-03-07

**Authors:** Zaher Merhi, Serin Seckin, Priscilla Morelli, Manasi Karekar, Marco Mouanness

**Affiliations:** 1 Department of Obstetrics and Gynecology, Division of Reproductive Endocrinology and Infertility, Maimonides Medical Center, Brooklyn, NY USA; 2 Reproductive Endocrinology and Infertility, Rejuvenating Fertility Center, New York, NY USA; 3 Department of Obstetrics and Gynecology, Division of Reproductive Endocrinology and Infertility, Columbia University Medical Center, New York, NY USA; 4 Florida State University College of Medicine, Tallahassee, FL USA

**Keywords:** COVID 19, Pandemic, Infertility treatment, Racial disparity, Black women

## Abstract

There are racial disparities in access and outcomes of assisted reproductive technology treatment in the USA; however, the effect of the pandemic on racial disparity within reproductive healthcare has not been extensively studied. This study aimed to identify how the pandemic has affected patient decision-making regarding fertility planning and treatment among Black *versus* non-Black women. The decision-making parameters that were assessed included discontinuing or changing the fertility treatment plans as well as visits to the clinic. This is a cross-sectional questionnaire study that was conducted at a university-affiliated fertility clinic between January and December 2021. A link to a survey was emailed to patients who were asked to fill out a questionnaire regarding fertility plans prior to and during the pandemic, in particular, the choice of the type of fertility treatment, exposure to COVID-19, and acceptability of the vaccine while trying to conceive, canceling or postponing the fertility treatment, and the use of telehealth during the pandemic. Out of 223 patients, the majority reported that the pandemic did not change their plans, and the minority reported either postponing or canceling their treatments with financial instability being the most reported reason. Fewer Black women were vaccinated compared to non-Black women. When asked whether the pandemic was well-handled by major healthcare systems, Black women were less likely than non-Black women to think that it met expectations. Additionally, Black women were less likely to be comfortable visiting fertility clinics in person and less interested in future at-home monitoring, if available, compared to non-Black women. Thus, among women undergoing fertility treatments during the pandemic, Black individuals were associated with a lower percentage of being vaccinated, a lower percentage of being satisfied with major healthcare systems handling the pandemic, and lesser comfort in visiting the fertility clinic physically. There is a clear need to understand the underlying reasons as to why the pandemic contributed to a racial disparity in fertility treatments.

## Introduction

Health disparities, defined as a higher burden of disease, illness, injury, disability, serious morbidity, or mortality as well as access to healthcare and resources among one group relative to another, are well documented in the USA [1]. One area where racial disparity has become prominent is access to and success of fertility treatments [2]. For example, Black women have two-fold higher odds of being affected by infertility, have less access to fertility care [3], and have significantly lower pregnancy rates by assisted reproductive technology such as in vitro fertilization (IVF), when compared to non-Black women [4].

The infection by severe acute respiratory syndrome coronavirus-2 (SARS-CoV-2) and its resultant coronavirus diseases-19 (COVID-19) pandemic have been shown to disproportionally affect minorities, in particular, Black women compared to Caucasians [5]. The COVID-19 pandemic has exacerbated these disparities by straining healthcare systems, limiting access to essential services, and disproportionately impacting vulnerable populations due to pre-existing inequities in health, economic stability, and social determinants of health [5]. Racial disparities in reproductive healthcare have been reflected by [1] accessibility such as access to healthcare, education, and affordable treatments and [2] by patients’ attitudes such as addressing trust in the healthcare system, motivations to seek infertility care, and changes in planning for fertility treatments. While the aspect of accessibility has been extensively studied, particularly in the context of the COVID-19 pandemic [6], whether there was a disparity in attitude towards fertility treatment during the pandemic between Black and non-Black individuals remains underexplored.

Existing research has examined how accessibility issues, including financial barriers, lack of education, and systemic inequities, contribute to racial disparities in fertility care and treatment outcomes. There is a well-established correlation between race and socioeconomic status, with minority populations often facing greater economic hardships. Black patients are less likely to have commercial insurance leading to significant disparities in telehealth utilization and presentation for an initial consultation in the African American population during the pandemic [7]. These financial challenges are compounded by the high cost of fertility treatments, which are often not covered by basic insurance plans. As a result, low-income couples face a more difficult financial decision when considering infertility treatments. The expense of these services can create a significant barrier to access, leaving many individuals unable to pursue the care they need in a timely manner.

Building on the economic barriers to fertility care, another significant issue is the lack of patient-centered education, particularly in Black communities. The pandemic led to many changes in insurance policies, but these changes may not have been communicated effectively, leaving many patients unaware of their options or how to navigate coverage [7]. Additionally, there is a disparity in knowledge about newer treatments and technologies, leaving many patients uninformed about the full range of options available to them. Black and lower-income populations, in particular, may not have the same access to online resources such as social media and websites, where many of these discussions take place [8]. While telehealth and other online healthcare resources became more prominent during the pandemic, access to these digital platforms remains uneven for certain communities. While telehealth use decreased the no-show rate overall, this change did not extend to African American patients, underscoring inequalities in insurance coverage and telehealth utilization. Even with expanded coverage for telehealth services, racial minorities, patients with limited English proficiency, and those with lower incomes continued to face barriers to accessing these services [9].

Although Black women are more likely to report infertility compared to their white counterparts, they are significantly less likely to pursue fertility treatments [10, 11]. This discrepancy may be partly due to the perception among Black women that infertility is rare within their community which can result in delays in seeking treatment [11]. Unfortunately, this delay can lead to reduced success rates, as age becomes a critical factor in fertility outcomes. Understanding these attitudes and motivations is essential to fully grasp the complexities of racial disparities in fertility care, especially in the context of the pandemic. The exact causes of the racial disparities in fertility care are not fully clear. During the pandemic, preliminary studies suggest a racial disparity in reproductive healthcare [6, 10, 11]; however, whether there was a disparity in attitude towards fertility treatment, in particular the choice of the type of fertility treatment, exposure to COVID-19 and acceptability of the vaccine while trying to conceive, canceling or postponing the fertility treatment, and the use of telehealth during the pandemic between Black and non-Black individuals have not been fully explored. Thus, we hypothesize that Black individuals have different attitudes towards fertility treatment and the healthcare system compared to non-Black individuals.

## Materials/Methods

### Data Collection

This is a cross-sectional questionnaire study that was conducted at a university-affiliated fertility clinic. Inclusion criteria included new and returning healthy infertility patients and women contemplating oocyte freezing for fertility preservation between January 2021 and December 2021. Men and women, who were already pregnant following infertility treatment during that period of time, were excluded from the study. Participants who did not fill out the question about their race were also excluded. Following the approval of the Institutional Review Board, a link to a survey was sent to the emails of all participants presenting to the fertility clinic.

The questionnaire was emailed three times to each participant: in mid-January 2021, mid-February 2021, and mid-April 2021. Each participant was asked to fill out a questionnaire that included several parts: (1) questions pertaining to demographics that included: age, race, relationship status, sexual identity, parity, and employment status; (2) items related to the type of fertility treatment the participant was planning to undergo such as intrauterine insemination (IUI), in vitro fertilization (IVF), oocyte freezing, or just a general infertility evaluation; (3) questions pertaining to the exposure to COVID-19 and acceptability of the vaccine, in particular whether the participant thought that the vaccine could negatively impact her chances of getting pregnant; (4) questions pertaining to her attitudes towards the fertility treatment during the pandemic such as (i) whether she changed or postponed (short-term, i.e., in the next 3 months, or long-term, i.e., for more than 3 months) her fertility treatment plans, (ii) her comfort visiting fertility clinics in person, (iii) reasons why the pandemic made her cancel/postpone her fertility treatment, and (iv) how she felt the pandemic was being handled by the healthcare system and fertility clinics in the USA as a whole; and, finally, (5) questions pertaining to her preferences and future vision for fertility care in the USA, and in particular (i) whether she prefers in person or a telehealth only consultations, (ii) whether she foresees a future for potentially undergoing “at home monitoring system” such as measuring her blood hormone levels and having a transvaginal ultrasound at home would become an option, and (iii) whether she foresees a future where mobile fertility clinics coming to her house for monitoring and undergoing all fertility procedures (such as IUI, oocyte collection, and embryo transfer) on a mobile unit would become a future option.

### Statistical Analysis

Continuous data between Black and non-Black participants were presented as mean ± standard error of the mean (SEM). Sample size calculation was performed [12] with the main outcome being the percentage of women “comfortable to visit fertility clinics during the pandemic.” In order to detect a 50% difference between Black versus non-Black and with a power of 80% and alpha of 0.05, 36 participants would be needed in each group of the study. Categorical data in both groups were summarized as counts and percentages. Normal distribution of continuous data was evaluated using Shapiro–Wilk test. *T*-test and chi-square tests were used for comparing continuous and categorical data, respectively. Multivariate logistic regression analysis was conducted to identify independent correlates. GraphPad 9.0 was used for statistical analyses, and *p* < 0.05 was considered statistically significant.

## Results

The questionnaire was emailed to 1257 patients, of which 223 (17.7%) participants (mean age 41 ± 1.6) filled it out. Participants whose answers were “others” (*n* = 21) and “prefer not to answer” (*n* = 16) to any specific question were excluded from the statistical analysis of that answer’s data. Table 1 represents the demographics and clinical characteristics of both Black (*n* = 49) and non-Black (*n* = 137) participants. The sample size of each race subcategory in the non-Black participants were Caucasian (*n* = 111), Asian (*n* = 14), South Asian (*n* = 7), American Indian (*n* = 1), Middle Eastern (*n* = 3), and Native Hawaiian (*n* = 1).

**Table 1:** Demographics, clinical characteristics, and responses to the questionnaire of Black and non-Black participants

Parameters^a^	Black (*n* = 49)	Non-Black (*n* = 137)
Average age	42.1 ± 6.6	40.5 ± 6.2
**Relationship status** - Married - Single - Living with partner - Not living with a partner - Divorced	24 (48.9) 15 (30.6) 8 (16.3) 1 (2) 1 (2)	108 (78.8) 9 (6.6) 14 (10.2) 2 (1.5) 4 (2.9)
**Sexual identity** - Heterosexual - Lesbian - Asexual - Bisexual - None of the above - I prefer not to answer	42 (85.7) 3 (6.1) 1 (2) 1 (2) 0 (0) 2 (4.1)	123 (89.8) 3 (2.2) 3 (2.2) 1 (0.7) 2 (1.5) 5 (3.6)
**Employment status** - Employed full time - Employed part-time - Currently or previously unemployed due to pandemic - Unemployed - Full or part time student	37 (75.5) 7 (14.3) 4 (8.1) 1 (2) 0 (0)	100 (73.0) 13 (9.5) 5 (3.6) 18 (13.1) 1 (0.7)
**Did you test positive for COVID-19 at any time?** - Yes - No	5 (10.2) 44 (89.8)	16 (11.7) 121 (88.3)
**Previous pregnancies** - 0 - 1 - 2 - 3 - 4 - 5 +	20 (40.8) 12 (24.5) 4 (8.1) 3 (6.1) 6 (12.2) 4 (8.1)	64 (46.7) 22 (16.1) 16 (11.7) 18 (13.1) 10 (7.3) 7 (5.1)
**Previous children** - 0 - 1 - 2 - 3 - 4 - 5 +	34 (69.4) 7 (14.3) 5 (10.2) 1 (2) 1 (2) 1 (2)	92 (67.1) 30 (21.9) 12 (8.7) 2 (1.5) 1 (0.7) 0 (0)
**Type of treatment prior to pandemic start** - Consultation only - Oocyte freezing - Timed intercourse - Embryo banking - IUI - IVF - Ovarian rejuvenation - Other - No answer	4 (8.1) 7 (14.3) 0 (0) 0 (0) 2 (4.1) 25 (51.0) 4 (8.1) 2 (4.1) 5 (10.2)	9 (6.6) 7 (5.1) 7 (5.1) 3 (2.2) 6 (4.4) 72 (52.6) 13 (9.5) 12 (8.7) 8 (5.8)
**Did your decision change due to the pandemic?** - No - No, but I postponed my plans from the earlier stages of the pandemic during the lockdown - Yes, I am canceling or have canceled my fertility plans - Yes, I am changing or have changed my fertility plan to a different procedure /treatment/method - Yes, I am postponing or postponed my fertility plans during the last year - No answer	30 (61.2) 6 (12.2) 1 (2) 3 (6.1) 4 (8.1) 5 (10.2)	89 (64.9) 23 (16.8) 2 (1.5) 4 (2.9) 11 (8.0) 8 (5.8)
**Comfort visiting fertility clinic during the pandemic** - Neutral - Slightly comfortable - Slightly/very uncomfortable* - Very comfortable - Prefers not to answer	9 (18.4) 8 (16.3) 3 (6.1) 24 (48.9) * 5 (10.2)	17 (12.4) 18 (13.1) 23 (16.8) 71 (51.8) * 8 (5.8)
**Reason why pandemic made you cancel your fertility treatment** - Financial instability* - Fetal health - Maternal health - Fear of attending office visits - Age - Frustration with travel - Other - No answer	14 (28.6) * 3 (6.1) 4 (8.1) 1 (2) 1 (2) 1 (2) 1 (2) 24 (48.9)	4 (2.9) * 8 (5.8) 10 (7.3) 13 (9.5) 0 (0) 19 (13.9) 17 (12.4) 66 (48.2)
**How do you feel the pandemic was being handled in the health care system as a whole** - Met expectation* - Exceeded expectation - Below Expectation - No answer	16 (32.6) * 9 (18.4) 13 (26.5) 11 (22.4)	70 (51.1) * 24 (17.5) 28 (20.4) 15 (10.9)
**Planning on receiving the vaccine** - Already vaccinated* - No - Unsure - Yes - Blank	16 (32.6) * 8 (16.3) 7 (14.3) 7 (14.3) 11 (22.4)	67 (48.9) * 20 (14.6) 19 (13.9) 17 (12.4) 14 (10.2)
**Fear of effect of vaccine on fertility?** - No effect - Negative effect on oocytes - Negative effect on implantation - Negative effect on sperm	17 (34.7) 15 (30.6) 16 (32.6) 6 (12.2)	38 (27.7) 40 (29.2) 41 (29.9) 18 (13.1)
**Do you think the covid vaccine can negatively impact pregnancy if taken while pregnant?** - Yes - Unsure - No - No answer	12 (24.5) 17 (34.7) 9 (18.4) 11 (22.4)	32 (23.4) 61 (44.5) 30 (21.9) 14 (10.2)
**If you chose to do the vaccine, did you stop or paused your fertility treatment for the covid vaccine** - Yes - No - Received vaccine while pregnant - Received vaccine prior to fertility treatment - Blank - Other	3 (6.1) 23 (46.9) 3 (6.1) 6 (12.2) 14 (28.6) 0 (0)	12 (8.7) 58 (42.3) 8 (5.8) 28 (20.4) 15 (10.9) 16 (11.7)
**In person or telehealth?** - No preference - Telehealth - In person - Blank	9 (18.4) 6 (12.2) 23 (46.9) 11 (22.4)	31 (22.6) 34 (24.8) 56 (40.8) 16 (11.7)
**Would you utilize at-home monitoring? (blood and US)** - Yes - No* - Blank	35 (71.4) 3 (6.1) * 11 (22.4)	90 (65.7) 31 (22.6) * 16 (11.7)
**Van for monitoring?** - Yes - No - Blank	34 (69.4) 4 (8.1) 11 (22.4)	98 (71.5) 22 (16.1) 17 (12.4)

^a^Data are expressed as mean ± SEM; all other data are expressed as *N* (%)

Chi-square test and *t*-test were used for comparing continuous and categorical data, respectively

^*^p < 0.05

When all participants, Black and non-Black combined, were asked about fertility treatment plans before the pandemic, 58% were pursuing IVF, 7% were planning oocyte freezing, 4% were planning IUI, and 31% were having infertility workup. The majority (69%) reported that the pandemic did not change their plans while the minority (31%) reported either totally changing (4%, *p* = 0.02), postponing for short-term (15%, *p* = 0.02), postponing for long-term (10%, *p* = 0.03), or forever canceling (2%, *p* = 0.03) their treatments with financial instability being the most reported reason (22%).

When comparing Black to non-Black participants, there was no statistical difference between age, relationship status, sexual identity, employment status, parity, or number of previous pregnancies (*p* > 0.05; Table 1). The type of fertility treatment that was planned before the pandemic started was also not statistically significant between Black and non-Black participants (*p* > 0.05).

Fewer Black participants were vaccinated compared to non-Black counterparts (32.6% vs. 48.9%; *p* = 0.03). When asked whether the pandemic was well-handled by major healthcare systems including fertility clinics in the USA, Black participants were less likely to think that it met expectations compared to non-Black participants (32.6% vs. 51.1%; *p* = 0.02) and more likely to report that financial instability was the reason to discontinue any treatment (28.6% vs. 2.9%; *p* < 0.0001). Additionally, Black participants were less likely to be comfortable (i.e., those who responded by saying “slightly uncomfortable” or “very uncomfortable”) visiting the office in person during the pandemic compared to non-Black participants (6.1% vs. 16.8%; *p* = 0.02) but not more likely to use telehealth compared to non-Black participants (12.2% vs. 24.8%; *p* = 0.07). There was no difference between Black and non-Black participants when asked whether they think that the vaccine could affect fertility or pregnancy outcomes or whether taking the vaccine stopped them from pursuing fertility treatments (*p* > 0.05). Both Black and non-Black participants equally foresee a future for potentially implementing mobile fertility clinics coming to the house for monitoring and undergoing all fertility procedures on the mobile unit (*p* > 0.05); however, Black participants were less likely to undergo “at-home monitoring with blood and ultrasounds” (*p* = 0.009) if it became available. All the results remained statistically significant after adjusting for age using multivariate logistic regression.

## Discussion

While it is known that racial disparity exists in fertility care inside and outside [13] the USA, this study reports novel disparity-related concerns that the COVID-19 pandemic unveiled between Black and non-Black women seeking fertility treatments. Black women were less likely to be comfortable going in person to fertility clinics during the pandemic which seems to have a long-lasting presence in reproductive endocrinology and infertility (REI) [7]. The experiences of underprivileged women within the healthcare system might suggest that trust in the system plays a role in fertility-related decision-making. The trust that patients hold in healthcare has declined over the past half-century [14], and it is even lower among Black communities, as confirmed by our results, which have experienced disproportionate healthcare access, and possible racism within the healthcare system. Evidence-based strategies to building trust within Black communities necessitate a commitment to health equity [15] and the use of research to measure the progress towards equity.

The COVID-19 pandemic has significantly impacted reproductive health, exacerbating existing disparities across different races and socioeconomic groups [16]. Access to contraception and abortion services became more challenging due to increased barriers, likely leading to a rise in unintended pregnancies. Underrepresented minorities and vulnerable populations faced disproportionate effects on their reproductive health, both from the virus itself and the resulting healthcare disruptions [7]. Additionally, initial uncertainties and misinformation regarding the safety of COVID-19 vaccines for pregnant and lactating women contributed to hesitancy [17]. Subsequent research has led major medical societies to recommend vaccination for women, regardless of pregnancy or lactation status [17, 18]. Addressing these issues is crucial for developing strategies to mitigate negative reproductive health outcomes during the current and future pandemics. A study [11] examined whether Black women experience poorer IVF outcomes compared to women of other races and sought to identify contributing factors. The results found that Black women had lower live birth rates and higher miscarriage rates following IVF [11]. These disparities persisted even after adjusting for variables such as age, body mass index, and the number of embryos transferred. That study suggested that underlying health conditions, socioeconomic factors, and potential biases within the healthcare system may contribute to these inequities, highlighting the need for further investigation and targeted interventions to improve IVF outcomes for Black women. Another study [7] examined the impact of the COVID-19 pandemic on initial infertility consultations, focusing on racial disparities in telehealth utilization. That retrospective cohort study found that, following the onset of the pandemic, there was a significant increase in telehealth use for initial infertility consultations. However, African American patients were less likely to utilize telehealth services compared to patients of other races. This disparity suggested that the shift to telehealth may exacerbate existing inequalities in access to fertility care, highlighting the need for targeted interventions to ensure equitable access to telehealth services across all racial groups.

Despite its merits by adding to a growing body of literature deepening the racial disparity in REI and family planning, this study has several limitations. First, we included participants from one IVF unit that has several satellite units in the Northeast of the USA which may limit the generalizability of these findings. Second, even though the questionnaire was emailed three times to a relatively large number of participants, the response rate was very low, which was not surprising given how the pandemic affected the daily life and the mental health of the general population. Emailing the participant could have been an access issue because maybe potential respondents from the clinic might not have had access to their emails especially during the pandemic [19]. It is possible that only individuals with a particular interest and/or strong opinion of COVID-19 and its impact on their attitude responded to the survey, thus introducing reporting bias. Third, the cross-sectional design of our study does not allow the assessment of causality or comparison to characteristics of infertile women before and after the availability and acceptability of the COVID-19 vaccine in the USA. A further limitation was that the questionnaire was sent via e-mail, thus only women feeling comfortable with or having access to this platform were able to participate.

## Conclusion

In conclusion, larger multi-center, multi-diverse, and multi-regional studies pertaining to the attitudes of Black and non-Black women “before, during, and after” the pandemic are crucial for reproductive endocrinologists, major healthcare systems, and governmental bodies to increase awareness and alter policies towards minimizing racial disparity in REI.

## Data Availability

Available upon request.
